# Smoking Status and Cognitive Function in a National Sample of Older Adults

**DOI:** 10.3389/fpsyt.2022.926708

**Published:** 2022-07-06

**Authors:** Qiaoyang Zhang, Min Zhang, Yun Chen, Shumin Zhu, Wang Zhou, Lihao Zhang, Guanzhong Dong, Yin Cao

**Affiliations:** ^1^Department of Psychology, The Affiliated Changzhou No. 2 People's Hospital of Nanjing Medical University, Changzhou, China; ^2^Department of Neurology, The Affiliated Changzhou No. 2 People's Hospital of Nanjing Medical University, Changzhou, China

**Keywords:** cigarette smoking, NHANES, older adults, cognitive function, processing speed

## Abstract

**Aims:**

To examine the correlation between smoking status and different domains of cognitive function in elderly Americans.

**Methods:**

We used data from the 2011 to 2014 U.S. National Health and Nutrition Examination Survey (NHANES). Participants over 60 years with available smoking history and cognitive function data were enrolled in our analysis. The NHANES study included the Consortium to Establish a Registry for Alzheimer's Disease (CERAD) assessment, the Animal Fluency Test (AFT), and the Digit Symbol Substitution Test (DSST) to assess cognition. Multivariate regression analyses were used to estimate the association between cigarette smoking and cognitive function.

**Results:**

A total of 2,932 participants were enrolled in the analysis, including 372 (12.7%) current smokers, 1,115 (38%) former smokers, and 1,445 (49.3%) never smokers. Never smokers had in average 3.82 (95% CI, 2.21 to 5.43) points more than current smokers in the DSST, whereas former smokers had 3.12 (95% CI, 1.51 to 4.73) points more than current smokers. Besides, smoking was not associated with the results of the AFT or the CERAD test.

**Conclusions:**

This study suggests that cigarette smoking is associated with processing speed among the American elderly.

## Introduction

Due to the marked aging of the world's population, the incidence of dementia has increased rapidly. The World Alzheimer Report 2015 predicted that nearly 131.5 million people will have dementia by 2050, which represents an enormous societal challenge (1). To date, there is no effective treatment for dementia.

As one of the biggest global public health challenges, tobacco is a potential lifestyle related to dementia. The evidence from epidemiological and experimental studies suggests that smoking may increase the risk of dementia and cognitive decline (2–10). In contrast, it appears that quitting smoking at any age reduces dementia risk. Researchers have found that smoking cessation for more than 4 years substantially reduces dementia risk over the next 8 years (HR 0.9, 95% CI 0.7 to 1.0) (11). Thus, the guideline for preventing dementia suggests avoiding smoking initiation and encourages smoking cessation (12).

Considering that cognitive dysfunction precedes dementia, it is essential to investigate the relationship between smoking and cognitive function. Researchers have found that small doses of nicotine have a short-time beneficial effect on cognitive performance, while long-term smoking leads to cognitive impairment (13). Besides, several studies have reported that quitting smoking has a beneficial effect on cognition in late life (7, 14, 15). However, little is known about the association of cigarette smoking and different domains of cognitive function in the American elderly. Therefore, this study aimed to examine the correlation between cigarette smoking and cognitive function across a range of domains.

## Materials and Methods

### Study Population

The NHANES is an ongoing survey designed by the U.S. National Center for Health Statistics (NCHS) to assess adults' and children's health and nutritional status. The NCHS Research Ethics Review Board approved the NHANES protocol, and all participants provided informed consent. More details of the NHANES data or the survey design are available from the center's official website (https://wwwn.cdc.gov/nchs/nhanes/tutorials/default.aspx).

**Figure 1:**
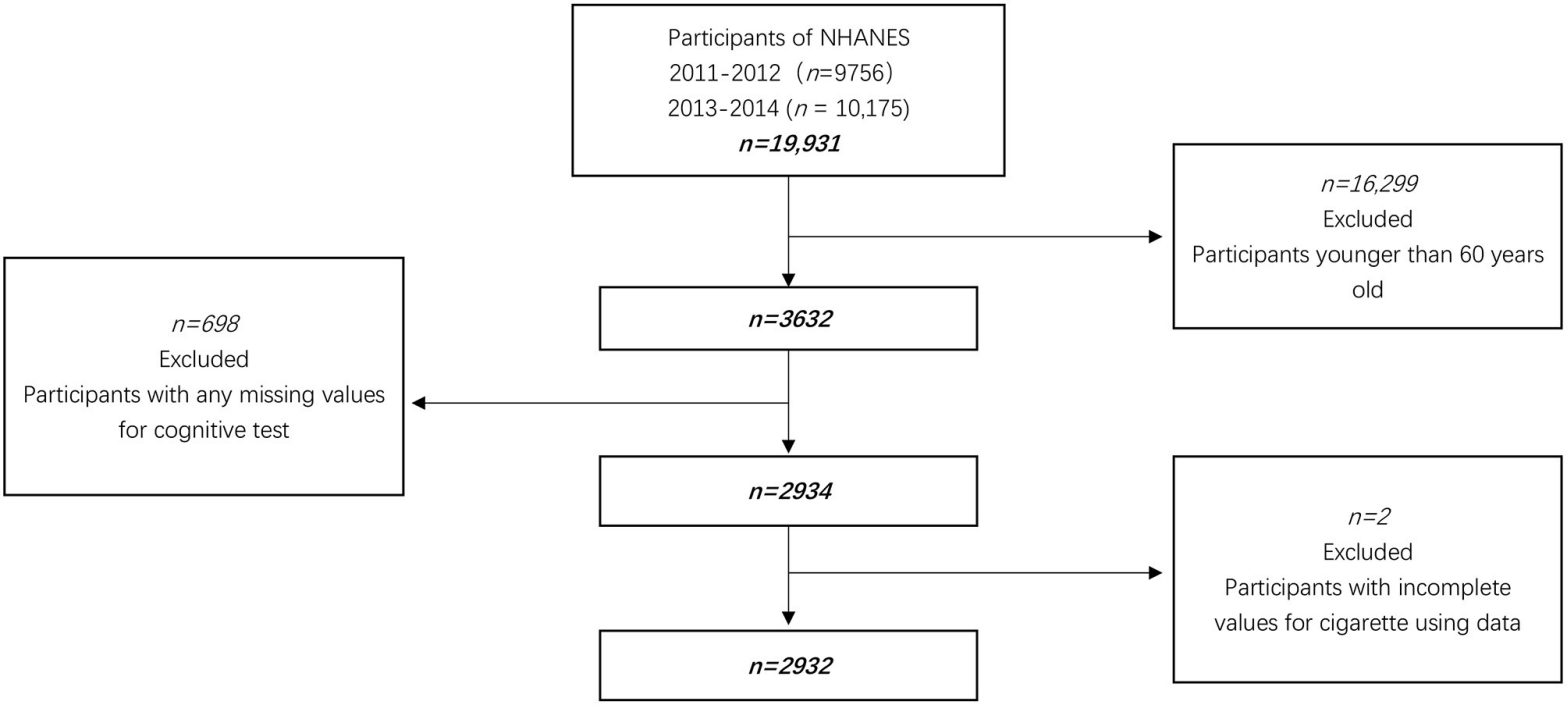
Flow chart of the enrolled participants. The bold values are the number of participants.

We combined 4 years of data (2011–2014) for this study. In our research, 3,632 aged 60 years and older were eligible because cognitive assessments were only administered in this age range. In addition, 2934 subjects completed all domains of cognitive tests. After further excluding participants with the incomplete value for cigarette using data, 2,932 participants were enrolled in this survey (Figure 1).

### Cigarette Smoking Habits

Participants were classified as never smokers (never smoked or smoked <100 cigarettes in life), former smokers (smoked more than 100 cigarettes in life and quit smoking), and current smokers (smoked more than 100 cigarettes in life and are currently smoking). Among the former smokers, 45 cases reported the time since smoking cessation in days/weeks/months, and we then transformed this information into years.

### Assessment of Cognitive Function

For the NHANES study, cognitive function was assessed with the Word learning and recall modules developed by the Consortium to Establish a Registry for Alzheimer's Disease (CERAD), the Animal Fluency Test (AFT), and the Digit Symbol Substitution Test (DSST) (16, 17).

The CERAD Word Learning subtest (CERAD W-L) is designed to assess immediately and delayed learning abilities for new verbal information, a component of the memory sub-domain. The test consists of three consecutive learning trials (Immediate word recall test, IWRT) and one delayed recall trial (Delayed word recall test, DWRT). For the learning trials, participants read out loud 10 unrelated words and then had to recall immediately as many words as they could. The order of the ten words changed for each trial. After completing the other cognitive tests (AFT and DSST), the delayed word recall measure was administered.

During the AFT, participants had to name as many animals as possible within a minute. Participants earned points for each correctly named animal. A practice test in which participants were asked to name three items of clothing was first conducted in the NHANES before the main test was completed.

The DSST is a reliable instrument to evaluate processing speed and executive function (18). Participants were given a piece of paper with a key that paired numbers and symbols. Next, they were asked to draw the corresponding symbols adjacent to the numbers within 2 min. The score was the total number of correct matches, ranging from 0 to 133.

### Covariates

According to previous research studies, we investigated a group of potential confounding factors, which included sex, age, ethnicity, education, marital status, body mass index (BMI), drinking habits (as measured by the question “Have you had at least 12 alcoholic drinks of any type in any given year?”), physical activity, depression (9-item Patient Health Questionnaire), and self-reported history of diseases (hypertension, diabetes, coronary heart disease, and stroke).

### Statistical Analysis

Primary sampling units (clusters), strata, and sampling weights were considered in the analyses to account for the complex sampling design of the NHANES. The original sample weight WTMEC2YR divided by two was used as the new sample weight because we combined two cycles of the NHANES data (19). Weighted mean (SE) was calculated to describe continuous variables, and weighted frequencies (%) (SE) were used to express categorical variables. The baseline characteristics of different groups of smoking status were analyzed using one-way ANOVA (normal distribution), non-parametric tests (in cases of non-normal distribution), and the chi-square test (for categorical variables). The cognitive function score was normally distributed and did not require transformation. We used multivariable linear regression models to examine the relationship between smoking and cognitive function. Model 1 was adjusted for sex, age, ethnicity, education, and marital status. Model 2 was additionally adjusted for BMI, drinking, physical activity, and depression severity. Model 3 was adjusted for model 2 plus history of diseases (hypertension, coronary heart disease, stroke, diabetes). The potential confounders were in line with the literature (7, 20).

Additionally, we ran a multivariable logistic regression analysis between smoking and low DSST performance to demonstrate its practical significance. DSST scores were stratified by age groups (60–69, 70–79, ≥80), then the lowest quartile of each group was used as a cut-off to define low DSST performance. Accordingly, the cut-off values were 37, 31, and 27 for the three age groups. This approach was consistent with the previously published literature (21).

All analyses were performed with the statistical software package R 3.3.2 (http://www.R-project.org, The R Foundation). The level of significance was defined as *P* < 0.05 (2-sided).

## Results

### Baseline Characteristics of Participants

Table 1 shows the participants' characteristics according to smoking status. A total of 2,932 participants were analyzed in this study, including 372 (12.7%) current smokers, 1,115 (38%) former smokers, and 1,445 (49.3%) never smokers. Never smokers were more likely to be women, more educated, and free of stroke or coronary heart disease. In contrast, current smokers were more likely to be younger, thinner, less educated, have lower intensity of recreational activity, more depressed, and current drinkers. Former smokers were more likely to have suffered from hypertension or diabetes than both current and never smokers.

**Table 1:** Weighted characteristics of participations, NHANES 2011–2014 (*N* = 2,932).

	**All**	**Current smokers**	**Former smokers**	**Never smokers**	***P*-value**
Unweighted *N*	2,932	372	1,115	1,445	
Age, median (IQR)	68.0 (11.0)	65.0 (12.0)	69.0 (12.0)	68.0 (12.0)	<0.001
Gender, n (%)					<0.001
Male	1,427 (45.6)	217 (51.5)	694 (56.2)	516 (35.8)	
Female	1,505 (54.4)	155 (48.5)	421 (43.8)	929 (64.2)	
Race, n (%)					<0.01
Hispanic American	553 (7.1)	78 (8.6)	189 (6.1)	286 (7.5)	
Non-Hispanic White	1,401 (79.5)	136 (68.8)	598 (83.5)	667 (78.7)	
Non-Hispanic Black	697 (8.4)	136 (13.9)	240 (6.9)	321 (8.4)	
Other Race	281 (5.0)	22 (8.7)	88 (3.6)	171 (5.4)	
Education, n (%)					<0.001
Less than high school	745(16.0)	126 (23.2)	281 (16.4)	338 (14.0)	
High school	686 (22.2)	96 (28.7)	277 (22.4)	313 (20.5)	
College or higher	1,498 (61.9)	150 (48.1)	556 (61.2)	792 (65.5)	
Marital status, n (%)					0.095
Married/Cohabitation	1,692 (64.7)	179 (58.1)	680 (66.3)	833 (64.9)	
Was married	1,070 (30.9)	165 (35.8)	384 (30.6)	521 (30.2)	
Never married	166 (4.3)	28 (6.1)	51 (3.2)	87 (4.9)	
Had at least 12 alcoholic drinks/year, n (%)	1,960 (72.7)	311 (87.5)	922 (85.8)	727 (59.0)	<0.001
Moderate to vigorous work activity, n (%)	883 (34.5)	117 (36.4)	352 (36.9)	414 (32.1)	0.485
Moderate to vigorous recreational activity, n (%)	1,218 (44.7)	106 (26.0)	483 (48.1)	633 (46.2)	<0.001
History of diseases, n (%)					
Hypertension	1,829 (59.1)	217 (53.5)	713 (62.2)	899 (57.8)	0.084
Diabetes	688 (19.4)	82 (18.0)	297 (22.5)	309 (17.3)	0.114
Coronary heart disease	266 (9.4)	39 (14.6)	122 (10.9)	105 (7.2)	<0.01
Stroke	203 (6.4)	37 (9.1)	87 (7.5)	79 (5.0)	<0.05
BMI, kg/m^2^, median (IQR)	28.0 (7.5)	26.2 (7.9)	28.9 (7.7)	27.8 (6.9)	<0.001
Depression, median (IQR)	1.0 (4.0)	2.0 (4.0)	1.0 (4.0)	1.0 (4.0)	0.496
AFT, mean (SE)	18.1 (5.7)	17.6 (5.5)	18.0 (5.5)	18.2 (5.9)	0.492
DSST, mean (SE)	51.9 (16.8)	48.3 (16.1)	51.3 (16.2)	53.3 (17.3)	<0.01
DWRT, mean (SE)	6.2 (2.3)	6.2 (2.1)	6.2 (2.2)	6.3 (2.4)	0.655
IWRT, mean (SE)	19.7 (4.5)	19.5 (4.2)	19.6 (4.3)	19.9 (4.7)	0.148

*Data presented are mean (S.E.), median (IQR), or n (%)*.

*S.E., Standard Error of Mean; IQR, Interquartile Range; BMI, Body Mass Index; AFT, Animal Fluency Test; DSST, Digit Symbol Substitution Test; DWRT, Delayed Word Recall Test; IWRT, Immediately Word Recall Test*.

### Univariate Linear Regression Analysis

The results showed that age, sex, ethnicity, education level, marital status, drinking, physical activity, depression, and history of diseases (hypertension, coronary heart disease, stroke, diabetes) were associated with cognitive function (see Supplementary Table S1 in the Appendix).

### Multivariate Linear Regression Analysis Between Smoking Status and Cognitive Function

Table 2 shows the association between smoking status and different domains of cognitive function, with current smokers being used as the reference group. In the fully adjusted model, never smokers had in average 3.82 (95% CI, 2.21 to 5.43) points more than current smokers in the DSST, whereas former smokers had 3.12 (95% CI, 1.51 to 4.73) points more than current smokers. Further, smoking status was not correlated with the scores of the AFT, DWRT, or IWRT, corresponding to different domains of cognitive function.

**Table 2:** Weighted multivariable linear regressions analysis between smoking status and cognitive function.

		**Crude model**	**Model 1**	**Model 2**	**Model 3**
		**β(95% CI)**	**β(95% CI)**	**β(95% CI)**	**β(95% CI)**
DSST score					
	Current smokers	Reference	Reference	Reference	Reference
	Former smokers	2.97 (0.9, 5.04)^**^	3.47 (1.86, 5.08)^***^	2.99 (1.37, 4.61) ^***^	3.12 (1.51, 4.73)^***^
	Never smokers	4.97 (2.95, 6.99)^***^	3.86 (2.28, 5.44)^***^	4.00 (2.39, 5.62) ^***^	3.82 (2.21, 5.43)^***^
AFT score					
	Current smokers	Reference	Reference	Reference	Reference
	Former smokers	0.45 (−0.25, 1.15)	0.4 (−0.23, 1.03)	0.1 (−0.55, 0.74)	0.12 (−0.53, 0.77)
	Never smokers	0.61 (−0.07, 1.3)	0.47 (−0.15, 1.09)	0.21 (−0.43, 0.86)	0.15 (−0.5, 0.79)
DWRT score					
	Current smokers	Reference	Reference	Reference	Reference
	Former smokers	−0.03 (−0.31, 0.25)	0.22 (−0.04, 0.49)	0.17 (−0.11, 0.44)	0.18 (−0.1, 0.45)
	Never smokers	0.09 (−0.19, 0.36)	0.1 (−0.16, 0.36)	0.01 (−0.26, 0.29)	0.03 (−0.25, 0.3)
IWRT score					
	Current smokers	Reference	Reference	Reference	Reference
	Former smokers	0.09 (−0.47, 0.64)	0.45 (−0.06, 0.96)	0.36 (−0.18, 0.89)	0.39 (−0.15, 0.93)
	Never smokers	0.41 (−0.13, 0.95)	0.28 (−0.22, 0.77)	0.24 (−0.29, 0.77)	0.23 (−0.31, 0.77)

*Model 1 adjusted for gender, age, race, education, marital status*.

*Model 2 adjusted for gender, age, race, education, marital status, BMI, drinking, physical activity, depression*.

*Model 3 adjusted for gender, age, race, education, marital status, BMI, drinking, physical activity, depression, and history of diseases (hypertension, coronary heart disease, stroke, diabetes)*.

** *P < 0.01*,

*** *P < 0.001*.

### Multivariate Linear Regression Analysis Between Smoking Cessation and DSST Test

Table 3 shows the association between smoking cessation and DSST test. In the fully adjusted model, former smokers who quitted <20 years before scored 2.39 (95% CI, 0.53 to 4.26) points higher than current smokers in the DSST, whereas subjects who quitted more than 30 years before had 5.45 (95% CI, 2.95 to 7.95) points more than current smokers.

**Table 3:** Weighted multivariable linear regression analysis between smoking cessation and DSST score.

	**No. of participants (N)**	**Crude model**	**Model 1**	**Model 2**	**Model 3**
		**β(95% CI)**	**β(95% CI)**	**β(95% CI)**	**β(95% CI)**
Current smokers	372	Reference	Reference	Reference	Reference
Former smokers					
quit <20 years	386	1.00 (−1.85, 3.85)	1.97 (−0.30, 4.25)	1.97 (−0.12, 4.06)	2.39 (0.53, 4.26)^*^
quit 20-30 years	384	3.94 (0.63, 7.25)^*^	4.15 (1.95, 6.34)^***^	4.14 (1.93, 6.36) ^***^	4.48 (2.41, 6.56)^***^
quit >30 years	345	4.59 (1.91, 7.27)^***^	6.21 (3.50, 8.92)^***^	5.35 (2.82, 7.88) ^***^	5.45 (2.95, 7.95)^***^
Never smokers	1,445	5.05 (2.32, 7.79)^***^	4.24 (1.48, 7.00)^**^	4.75 (2.24, 7.26) ^***^	4.78 (2.53, 7.04)^***^

*Model 1 adjusted for gender, age, race, education, marital status*.

*Model 2 adjusted for gender, age, race, education, marital status, BMI, drinking, physical activity, depression*.

*Model 3 adjusted for gender, age, race, education, marital status, BMI, drinking, physical activity, depression, and history of diseases (hypertension, coronary heart disease, stroke, diabetes)*.

* *P < 0.05*,

** *P < 0.01*,

*** *P < 0.001*.

### Multivariable Logistic Regression Analysis Between Smoking Cessation and Low DSST Performance

As shown in Table 4, in the fully adjusted model, the OR for low DSST performance was 0.46 (95% CI, 0.3 to 0.7) in former smokers who quit more than 30 years before compared to current smokers.

**Table 4:** Weighted multivariable logistic regression analysis between smoking cessation and low DSST performance.

	**No. of participants (N)**		**Crude model**	**Model 1**	**Model 2**	**Model 3**
	**Low**	**Normal**	**OR (95% CI)**	**OR (95% CI)**	**OR (95% CI)**	**OR (95% CI)**
Current smokers	132	240	Reference	Reference	Reference	Reference
Former smokers						
quit <20 years	108	278	0.71 (0.52, 0.97)^*^	0.74 (0.53, 1.04)	0.78 (0.54, 1.13)	0.78 (0.54, 1.13)
quit 20–30 years	102	282	0.57 (0.41, 0.79)^**^	0.57 (0.4, 0.83)^**^	0.71 (0.48, 1.05)	0.69 (0.47, 1.03)
quit >30 years	52	293	0.32 (0.22, 0.46)^***^	0.34 (0.23, 0.51)^***^	0.45 (0.29, 0.69) ^***^	0.46 (0.3, 0.7)^***^
Never smokers	331	1,114	0.54 (0.42, 0.69)^***^	0.59 (0.45, 0.79)^***^	0.63 (0.46, 0.86) ^**^	0.66 (0.48, 0.9)^**^

*Model 1 adjusted for gender, age, race, education, and marital status*.

*Model 2 adjusted for gender, age, race, education, marital status, BMI, drinking, physical activity, and depression*.

*Model 3 adjusted for gender, age, race, education, marital status, BMI, drinking, physical activity, depression, and history of diseases (hypertension, coronary heart disease, stroke, diabetes)*.

* *P < 0.05*,

** *P < 0.01*,

*** *P < 0.001*.

## Discussion

The findings of this population-based study indicated that smoking was negatively associated with processing speed in the American elderly. All the reported correlations remained significant after fully adjusting for confounding factors.

The results are in line with previous studies showing that smoking is related to cognitive impairment (22). A previous meta-analysis using data from 20 population-based cohorts reported that smoking is one of the modifiable factors correlated with late-life cognitive decline (6). Besides, a large German cohort study showed that the risk for cognitive decline in older subjects decreases with time after smoking cessation (7).

In the present study, one potential mechanism to explain the relationship between smoking and cognitive function is the inflammatory response, which may cause adverse effects on cognitive function (23–27). For example, a recent preclinical study reported that exposure to smoking exacerbates cognitive impairment in a rat model of vascular dementia through neuroinflammation (28). Another mechanism might involve psychosocial processes, such as sleep problems. A previous case-control study of mild cognitive impairment reported sleep duration partly mediates the connection between smoking and cognitive function (29). Functional and anatomic neuroimaging studies also have demonstrated several alterations in brain structural morphology and brain function to cigarette smoking. For example, a 24-month nonrandomized interventional study found that when compared with never smokers, current smokers have decreased gray matter density in areas critical to cognitive function (15). Accordingly, we speculate that the positive association between smoking cessation and cognition could also be due to the improvement of the above factors, but this possibility needs to be verified in future studies.

Regarding the different domains of cognition, contrary to some other studies indicating an association of smoking with greater risk of poor memory, we did not find an association between smoking and memory (15, 23). However, in the Lothian Birth Cohort 1936 study, the authors found that current smokers scored significantly lower than former smokers and never smokers on general cognitive ability and processing speed tests, but not memory or verbal ability (30). The inconsistency of the results may be related to ethnicity, age, and sample size. More research is needed in the future to detect the relationship between smoking and different domains of cognition.

This study exhibits several strengths. The connection between smoking and cognitive function was evaluated in a population-based sample of American older subjects. Thus, it investigated this relationship with a high statistical power. Additionally, we controlled for important confounding factors to precisely estimate the correlation between smoking and cognitive function.

## Limitations

There are several limitations of this study. First, the cross-sectional design could lead to a lack of causal relationship between smoking and cognitive function. Second, another potential limitation is that although we adjusted for a wide range of confounders, unmeasured biomarkers may have contributed to residual confounding.

## Conclusion

Using the NHANES database, the findings indicated that smoking was negatively associated with processing speed in the American elderly. Given the accelerated aging of the population and the high prevalence of smoking worldwide, our findings support the importance of tobacco control. High-quality, prospective studies are needed in the future to confirm our findings.

## Data Availability Statement

Publicly available datasets were analyzed in this study. These data can be found here: https://wwwn.cdc.gov/nchs/nhanes/tutorials/default.aspx.

## Ethics Statement

The studies involving humans were reviewed and approved by the National Center for Health Statistics Research Ethics Review Board. Participants provided their written informed consent to participate in the study.

## Author Contributions

MZ designed the study. QZ conducted the data analysis and wrote the manuscript. GD and YCa directed all the work. All authors contributed to the article and approved the submitted version.

## Funding

This study was supported by the General Program of Jiangsu Commission of Health (H2019051) and the Changzhou Health Care Young Talents Training Project (CZQM2020073).

## Conflict of Interest

The authors declare that the research was conducted in the absence of any commercial or financial relationships that could be construed as a potential conflict of interest.

## Publisher's Note

All claims expressed in this article are solely those of the authors and do not necessarily represent those of their affiliated organizations, or those of the publisher, the editors and the reviewers. Any product that may be evaluated in this article, or claim that may be made by its manufacturer, is not guaranteed or endorsed by the publisher.

## References

[1] Ijaopo EO. Dementia-related agitation: a review of non-pharmacological interventions and analysis of risks and benefits of pharmacotherapy. Transl Psychiatry. (2017) 7:e1250. 10.1038/tp.2017.199 29087372 PMC5682601

[2] Amini R Sahli M Ganai S. Cigarette smoking and cognitive function among older adults living in the community. Neuropsychol Dev Cogn B Aging Neuropsychol Cogn. (2021) 28:616–31. 10.1080/13825585.2020.1806199 32783580

[3] Tsai HJ Chang FK. Associations of exercise, nutritional status, and smoking with cognitive decline among older adults in Taiwan: Results of a longitudinal population-based study. Arch Gerontol Geriatr. (2019) 82:133–8. 10.1016/j.archger.2018.12.008 30784772

[4] Deal JA Power MC Palta P Alonso A Schneider ALC Perryman K. Relationship of cigarette smoking and time of quitting with incident dementia and cognitive decline. J Am Geriatr Soc. (2020) 68:337–45. 10.1111/jgs.16228 31675113 PMC7002272

[5] Wu J Dong W Pan XF Feng L Yuan JM Pan A. Relation of cigarette smoking and alcohol drinking in midlife with risk of cognitive impairment in late life: the Singapore Chinese Health Study. Age Ageing. (2019) 48:101–7. 10.1093/ageing/afy166 30307472 PMC6322505

[6] Lipnicki DM Makkar SR Crawford JD Thalamuthu A Kochan NA Lima-Costa MF. Determinants of cognitive performance and decline in 20 diverse ethno-regional groups: A COSMIC collaboration cohort study. PLoS Med. (2019) 16:e1002853. 10.1371/journal.pmed.1002853 31335910 PMC6650056

[7] Mons U Schöttker B Müller H Kliegel M Brenner H. History of lifetime smoking, smoking cessation and cognitive function in the elderly population. Eur J Epidemiol. (2013) 28:823–31. 10.1007/s10654-013-9840-9 23990211

[8] Cataldo JK Prochaska JJ Glantz SA. Cigarette smoking is a risk factor for Alzheimer's Disease: an analysis controlling for tobacco industry affiliation. J Alzheimers Dis. (2010) 19:465–80. 10.3233/JAD-2010-1240 20110594 PMC2906761

[9] Lee HJ Jang J Choi DW Chae W Park EC Jang SI. Association between change in lifestyle and cognitive functions among elderly Koreans: findings from the Korean longitudinal study of aging (2006-2016). BMC Geriatr. (2020) 20:317. 10.1186/s12877-020-01693-7 32867702 PMC7457530

[10] Wang Z Pang Y Liu J Wang J Xie Z Huang T. Association of healthy lifestyle with cognitive function among Chinese older adults. Eur J Clin Nutr. (2021) 75:325–34. 10.1038/s41430-020-00785-2 33116235

[11] Choi D Choi S Park SM. Effect of smoking cessation on the risk of dementia: a longitudinal study. Ann Clin Transl Neurol. (2018) 5:1192–9. 10.1002/acn3.633 30349854 PMC6186929

[12] Livingston G Huntley J Sommerlad A Ames D Ballard C Banerjee S. Dementia prevention, intervention, and care: 2020 report of the Lancet Commission. Lancet. (2020) 396:413–46. 10.1016/S0140-6736(20)30367-6 32738937 PMC7392084

[13] Almeida NL Rodrigues SJ Gonçalves LM Silverstein SM Sousa IC Gomes GH. Opposite effects of smoking and nicotine intake on cognition. Psychiatry Res. (2020) 293:113357. 10.1016/j.psychres.2020.113357 32823200

[14] Stewart MC Deary IJ Fowkes FG Price JF. Relationship between lifetime smoking, smoking status at older age and human cognitive function. Neuroepidemiology. (2006) 26:83–92. 10.1159/000090253 16352911

[15] Almeida OP Garrido GJ Alfonso H Hulse G Lautenschlager NT Hankey GJ. 24-month effect of smoking cessation on cognitive function and brain structure in later life. Neuroimage. (2011) 55:1480–9. 10.1016/j.neuroimage 21281718

[16] U. S. Centers for Disease Control Prevention. 2013–2014 Data Documentation, Codebook, and Frequencies: Cognitive Functioning (CFQ_H) 2017. Available online at: https://wwwn.cdc.gov/Nchs/Nhanes/2013-2014/CFQ_H.htm

[17] U. S. Centers for Disease Control Prevention. 2011–2012 Data Documentation, Codebook, and Frequencies: Cognitive Functioning (CFQ_G) 2017. Available online at: https://wwwn.cdc.gov/Nchs/Nhanes/2011-2012/CFQ_G.htm

[18] D'Amato CP Denney RL. The diagnostic utility of the Rarely Missed Index of the Wechsler Memory Scale-Third Edition in detecting response bias in an adult male incarcerated setting. Arch Clin Neuropsychol. (2008) 23:553–61. 10.1016/j.acn.2008.05.007 18585003

[19] Krieger N Williams DR Moss NE. Measuring social class in US public health research: concepts, methodologies, and guidelines. Annu Rev Public Health. (1997) 18:341–78. 10.1146/annurev.publhealth.18.1.341 9143723

[20] Lu Y Sugawara Y Zhang S Tomata Y Tsuji I. Smoking cessation and incident dementia in elderly Japanese: the Ohsaki Cohort 2006 Study. Eur J Epidemiol. (2020) 35:851–60. 10.1007/s10654-020-00612-9 32060675 PMC7525275

[21] Li S Sun W Zhang D. Association of Zinc, Iron, Copper, and Selenium intakes with low cognitive performance in older adults: a cross-sectional study from National Health and Nutrition Examination Survey (NHANES). J Alzheimers Dis. (2019) 72:1145–57. 10.3233/JAD-190263 31683474

[22] Anstey KJ von Sanden C Salim A O'Kearney R. Smoking as a risk factor for dementia and cognitive decline: a meta-analysis of prospective studies. Am J Epidemiol. (2007) 166:367–78. 10.1093/aje/kwm116 17573335

[23] Durazzo TC Mattsson N Weiner MW. Alzheimer's Disease Neuroimaging Initiative. Smoking and increased Alzheimer's disease risk: a review of potential mechanisms. Alzheimers Dement. (2014) 10:S122–45. 10.1016/j.jalz.2014.04.009 24924665 PMC4098701

[24] Kivipelto M Mangialasche F Ngandu T. Lifestyle interventions to prevent cognitive impairment, dementia and Alzheimer disease. Nat Rev Neurol. (2018) 14:653–66. 10.1038/s41582-018-0070-3 30291317

[25] Yaffe K Bahorik AL Hoang TD Forrester S Jacobs DR Jr Lewis CE. Cardiovascular risk factors and accelerated cognitive decline in midlife: the CARDIA study. Neurology. (2020) 95:e839–46. 10.1212/WNL.0000000000010078 32669394 PMC7605504

[26] Hay M Barnes C Huentelman M Brinton R Ryan L. Hypertension and age-related cognitive impairment: common risk factors and a role for precision aging. Curr Hypertens Rep. (2020) 22:80. 10.1007/s11906-020-01090-w 32880739 PMC7467861

[27] Suemoto CK Szlejf C Santos IS Brunoni AR Goulart AC Bertola L. Ideal vascular health and cognitive performance in the Brazilian longitudinal study of adult health. Eur J Neurol. (2021) 28:71–80. 10.1111/ene.14532 32920963

[28] Meng N Dong Y Huo T Song M Jiang X Xiao Y. Past exposure to cigarette smoke aggravates cognitive impairment in a rat model of vascular dementia via neuroinflammation. Cell Mol Neurobiol. (2022) 42:1021–34. 10.1007/s10571-020-00992-2 33156450 PMC11441291

[29] Hu M Yin H Shu X Jia Y Leng M Chen L. Multi-angles of smoking and mild cognitive impairment: is the association mediated by sleep duration? Neurol Sci. (2019) 40:1019–27. 10.1007/s10072-019-03750-5 30778881

[30] Corley J Gow AJ Starr JM Deary IJ. Smoking, childhood IQ, and cognitive function in old age. J Psychosom Res. (2012) 73:132–8. 10.1016/j.jpsychores.2012.03.006 22789417

