# Supplementary material for: Smoking Status and Cognitive Function in a National Sample of Older Adults
Source: Front Psychiatry. 2022 Jul 6;13:926708. doi: 10.3389/fpsyt.2022.926708 (PMC9301276; doi:10.3389/fpsyt.2022.926708)
Supplement: Supplementary file 1 [file Table_1.DOCX]

Appendix file

**Table S1: Weighed Univariate Analyses of cognitive tests.**

| Exposure | DSST score | | AFT score | | DWRT score | | IWRT score | |
| --- | --- | --- | --- | --- | --- | --- | --- | --- |
|  | β (95% CI) | P-value | β (95% CI) | P-value | β (95% CI) | P-value | β (95% CI) | P-value |
| Age | -1.01 (-1.1, -0.93) | < 0.001 | -0.26 (-0.29, -0.23) | < 0.001 | -0.11 (-0.13, -0.1) | < 0.001 | -0.22 (-0.24, -0.19) | < 0.001 |
| Gender |  | < 0.001 |  | 0.007 |  | < 0.001 |  | < 0.001 |
| Male | Reference |  | Reference |  | Reference |  | Reference |  |
| Female | 3.41 (2.2, 4.62) |  | -0.57 (-0.98, -0.16) |  | 0.59 (0.42, 0.75) |  | 1.3 (0.97, 1.62) |  |
| Race |  | < 0.001 |  | < 0.001 |  | < 0.001 |  | < 0.001 |
| Hispanic American | Reference |  | Reference |  | Reference |  | Reference |  |
| Non-Hispanic White | 16.64 (14.41, 18.87) |  | 2.79 (2, 3.58) |  | 0.72 (0.39, 1.04) |  | 2 (1.37, 2.64) |  |
| Non-Hispanic Black | 1.98 (-0.91, 4.88) |  | -0.93 (-1.95, 0.1) |  | 0.24 (-0.18, 0.66) |  | 1.21 (0.39, 2.03) |  |
| Other Race | 12.76 (9.46, 16.05) |  | -0.11 (-1.28, 1.06) |  | 0.81 (0.32, 1.29) |  | 1.01 (0.06, 1.95) |  |
| Education |  | < 0.001 |  | < 0.001 |  | < 0.001 |  | < 0.001 |
| Less than high school | Reference |  | Reference |  | Reference |  | Reference |  |
| High school | 12.47 (10.71, 14.24) |  | 1.64 (1.01, 2.28) |  | 0.56 (0.29, 0.83) |  | 1.83 (1.32, 2.35) |  |
| College or higher | 21.16 (19.65, 22.66) |  | 4.89 (4.34, 5.43) |  | 1.24 (1.01, 1.46) |  | 3.19 (2.75, 3.63) |  |
| Marital status |  | < 0.001 |  | < 0.001 |  | < 0.001 |  | < 0.001 |
| Married/Cohabitation | Reference |  | Reference |  | Reference |  | Reference |  |
| Was married | -5.73 (-7.04, -4.42) |  | -1.35 (-1.8, -0.9) |  | -0.35 (-0.53, -0.17) |  | -0.9 (-1.25, -0.55) |  |
| Never married | -2.96 (-5.9, -0.01) |  | -0.47 (-1.49, 0.54) |  | 0.03 (-0.38, 0.44) |  | -0.06 (-0.86, 0.75) |  |
| BMI | 0.02 (-0.08, 0.12) | 0.686 | 0.01 (-0.02, 0.05) | 0.389 | 0.02 (0.01, 0.03) | 0.007 | 0.02 (0, 0.05) | 0.109 |
| Have at least 12 alcoholic drinks/year |  | < 0.001 |  | < 0.001 |  | 0.003 |  | < 0.001 |
| NO | Reference |  | Reference |  | Reference |  | Reference |  |
| Yes | -6.33 (-7.68, -4.99) |  | -2.14 (-2.6, -1.68) |  | -0.29 (-0.48, -0.1) |  | -0.79 (-1.16, -0.43) |  |
| Work activity |  | < 0.001 |  |  |  | < 0.001 |  | < 0.001 |
| Vigorous activity | Reference |  | Reference |  | Reference |  | Reference |  |
| Moderate activity | -2.49 (-4.62, -0.37) |  |  |  | -0.19 (-0.48, 0.11) |  | -0.25 (-0.82, 0.33) |  |
| Other | -6.22 (-8.06, -4.38) |  |  |  | -0.48 (-0.74, -0.23) |  | -0.88 (-1.37, -0.38) |  |
| Recreational activity |  | < 0.001 |  | < 0.001 |  | < 0.001 |  | < 0.001 |
| Vigorous activity | Reference |  | Reference |  | Reference |  | Reference |  |
| Moderate activity | -8.71 (-10.73, -6.69) |  | -0.87 (-1.59, -0.14) |  | -0.59 (-0.87, -0.31) |  | -1.43 (-1.99, -0.88) |  |
| Other | -14.45 (-16.37, -12.53) |  | -1.87 (-2.49, -1.24) |  | -0.96 (-1.23, -0.69) |  | -2.24 (-2.77, -1.72) |  |
| PHQ-9 score | -0.75 (-0.89, -0.61) | < 0.001 | -0.18 (-0.24, -0.13) | < 0.001 | -0.03 (-0.05, -0.01) | 0.002 | -0.07 (-0.12, -0.03) | < 0.001 |
| History of hypertension |  | < 0.001 |  | < 0.001 |  | < 0.001 |  | < 0.001 |
| Yes | Reference |  | Reference |  | Reference |  | Reference |  |
| NO | 5.22 (4, 6.44) |  | 1.55 (1.13, 1.97) |  | 0.42 (0.25, 0.59) |  | 0.92 (0.59, 1.25) |  |
| History of diabetes |  | < 0.001 |  | < 0.001 |  | < 0.001 |  | < 0.001 |
| Yes | Reference |  | Reference |  | Reference |  | Reference |  |
| No | 8.48 (6.97, 9.99) |  | 1.63 (1.11, 2.15) |  | 0.49 (0.28, 0.7) |  | 1.05 (0.64, 1.46) |  |
| History of coronary heart disease |  | < 0.001 |  | < 0.001 |  | < 0.001 |  | < 0.001 |
| Yes | Reference |  | Reference |  | Reference |  | Reference |  |
| No | 5.98 (3.91, 8.05) |  | 1.2 (0.5, 1.91) |  | 0.59 (0.31, 0.88) |  | 1.13 (0.58, 1.69) |  |
| History of stroke |  | < 0.001 |  | < 0.001 |  | < 0.001 |  | < 0.001 |
| Yes | Reference |  | Reference |  | Reference |  | Reference |  |
| No | 10.92 (8.5, 13.34) |  | 2.64 (1.81, 3.48) |  | 0.7 (0.36, 1.04) |  | 1.61 (0.95, 2.27) |  |
